# Functional characterization of lysine-specific demethylase 2 (LSD2/KDM1B) in breast cancer progression

**DOI:** 10.18632/oncotarget.19387

**Published:** 2017-07-19

**Authors:** Lin Chen, Shauna N. Vasilatos, Ye Qin, Tiffany A. Katz, Chunyu Cao, Hao Wu, Nilgun Tasdemir, Kevin M. Levine, Steffi Oesterreich, Nancy E. Davidson, Yi Huang

**Affiliations:** ^1^ Women’s Cancer Research Center, UPMC Hillman Cancer Center, University of Pittsburgh School of Medicine, Pittsburgh, PA, USA; ^2^ School of Medicine, Tsinghua University, Beijing, P.R. China; ^3^ Department of Pharmacology & Chemical Biology, University of Pittsburgh School of Medicine, Pittsburgh, PA, USA; ^4^ Department of Pathology, University of Pittsburgh School of Medicine, Pittsburgh, PA, USA; ^5^ Center for Precision Environmental Health, Baylor College of Medicine, Houston, TX, USA; ^6^ Department of Oncology, The First Affiliated Hospital of Nanjing Medical University, Nanjing, Jiangsu, P.R. China; ^7^ Fred Hutchinson Cancer Research Center and Department of Medicine, University of Washington, Seattle, WA, USA; ^8^ China Three Gorges University, Yichang, Hubei, P. R. China

**Keywords:** LSD2/KDM1B, breast cancer, cell growth, migration, invasion

## Abstract

Flavin-dependent histone demethylases govern histone H3K4 methylation and act as important chromatin modulators that are extensively involved in regulation of DNA replication, gene transcription, DNA repair, and heterochromatin gene silencing. While the activities of lysine-specific demethylase 1 (LSD1/KDM1A) in facilitating breast cancer progression have been well characterized, the roles of its homolog LSD2 (KDM1B) in breast oncogenesis are relatively less understood. In this study, we showed that LSD2 protein level was significantly elevated in malignant breast cell lines compared with normal breast epithelial cell line. TCGA- Oncomine database showed that LSD2 expression is significantly higher in basal-like breast tumors compared to other breast cancer subtypes or normal breast tissue. Overexpression of LSD2 in MDA-MB-231 cells significantly altered the expression of key important epigenetic modifiers such as LSD1, HDAC1/2, and DNMT3B; promoted cellular proliferation; and augmented colony formation in soft agar; while attenuating motility and invasion. Conversely, siRNA-mediated depletion of endogenous LSD2 hindered growth of multiple breast cancer cell lines while shRNA-mediated LSD2 depletion augmented motility and invasion. Moreover, LSD2 overexpression in MDA-MB-231 cells facilitated mammosphere formation, enriched the subpopulation of CD49f^+^/EpCAM^-^ and ALDH^high^, and induced the expression of pluripotent stem cell markers, NANOG and SOX2. In xenograft studies using immune-compromised mice, LSD2-overexpressing MDA-MB-231 cells displayed accelerated tumor growth but significantly fewer lung metastases than controls. Taken together, our findings provide novel insights into the critical and multifaceted roles of LSD2 in the regulation of breast cancer progression and cancer stem cell enrichment.

## INTRODUCTION

Histone lysine methylation is an important covalent post-translational modification (PTM) of chromatin. Histone lysine methyltransferases (KMTs) and demethylases (KDMs) are groups of enzymes that have pivotal roles in dynamic regulation of numerous chromatin functions such as gene transcription, chromatin stability, DNA replication and repair [1, 2]. To date, two different classes of KDMs have been recognized: the flavin-dependent amine oxidase-containing and the Jumonji C (JmjC)-domain-containing enzymes. The flavin-dependent KDM family includes LSD1 (KDM1A) and LSD2 (KDM1B), which both contain a SWIRM domain and share significant sequence homology in their amine oxidase domains. However, LSD2 possesses an N-terminal zinc finger motif, which is required for binding to methylated histone lysine, while lacking LSD1’s co-factor binding tower domain. Both enzymes oxidize Carbon-Nitrogen bonds with subsequent production of a demethylated substrate, lysine 4 of histone 3, in a flavin-dependent manner [3, 4]. Although LSD1 and LSD2 are highly similar in amino acid sequences, catalyzed chemical reactions, and substrates, it is evident that the two enzymes also have distinct functions, and therefore may act differentially in regulating chromatin structure and function. Moreover, while LSD1 is mainly associated with the promoter region of genes, LSD2 tends to bind at transcribed coding regions and does not assemble the same transcription repressor complexes as LSD1 [5, 6]. These findings suggest that LSD1 and LSD2 likely interact with different protein partners in the nucleus and play quite distinct roles in regulating key cellular processes.

In the past decade, the flavin-dependent demethylase family has emerged as a potential therapeutic target for breast cancer. According to the data from The Cancer Genome Atlas (TCGA) database, mRNA expression levels of both LSD1 and LSD2 are greatly increased in breast cancer patient specimens in comparison to normal breast tissues. A role for LSD1 has been consistently implicated in tumorigenesis in various cancers, including breast cancer [7-14]. Importantly, LSD1 expression is highly associated with a more aggressive breast cancer phenotype, and work from our laboratory and others has consistently shown LSD1 depletion hinders proliferation and metastasis of breast cancer cells [8, 11, 15, 16]. Many small molecule inhibitors targeting LSD1 have been developed in the past years, and antineoplastic efficacy of several promising compounds has been tested in clinical trials for treatment of cancers such as acute myeloid leukemia (AML) and lung cancer (http://clinicaltrials.gov).

LSD2 has been linked to numerous important biological processes including transcription regulation, chromatin remodeling, genomic imprinting, heterochromatin silencing, growth factor signaling and somatic cell reprogramming [6, 17-20]. While the roles of LSD2 in breast cancer biology have been emerging, the underlying mechanisms are still largely unknown. Recent studies from our laboratory demonstrated that inhibition of LSD2 attenuates colony formation and downregulates global DNA methylation in breast cancer cells [21]. Combined inhibition of DNA methyltransferase (DNMT) and LSD2 reactivates expression of abnormally silenced genes with important functions in breast cancer and enhances cellular apoptotic responses. These findings suggest that combinatorial therapy targeting LSD2 and DNMTs effectively improves the antitumor efficacy of DNMT inhibitors in breast cancer. In this report, we elucidate the *in vitro* and *in vivo* activities of LSD2 in regulation of breast cancer proliferation, migration, invasion and cancer stem cell propagation. These studies provide novel insight into the multifaceted roles of LSD2 in breast cancer progression.

## RESULTS

### LSD2 expression is elevated in breast cancer cell lines and clinical specimens

**Figure 1:**
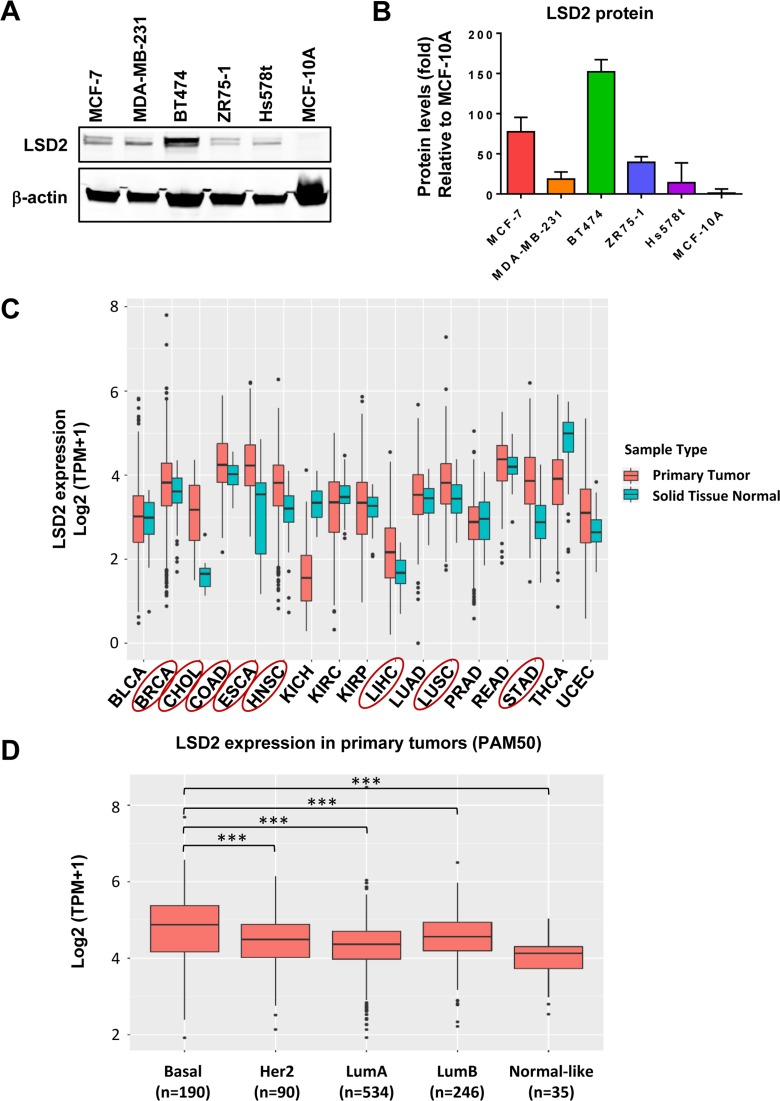
Expression level of LSD2 in breast cancer cell lines and clinical tumor specimens **A.** Western blot examination of LSD2 protein expression in breast cancer and MCF10A cell lines. **B.** Quantification of western blot results of LSD2 expression. **C.** TCGA data analysis of mRNA level of LSD2 in different types of cancer. Cancer types with significantly elevated LSD2 mRNA level were highlighted with Red circle. P-values were calculated using Mann-Whitney U test and corrected for multiple comparisons using Benjamini-Hochberg. **D.** mRNA levels of LSD2 in different subtypes of breast cancer. Tukey multiple comparisons of means, *** *p* < 0.001.

We examined LSD2 protein level in several human breast cancer cell lines and the normal immortalized human mammary epithelial cell line, MCF10A. Western blots showed that LSD2 protein expression is elevated in breast cancer cell lines compared with MCF10A cells (Figure 1A and 1B). Next, *in silico* analysis of LSD2 expression in clinical cancer patient samples indicated that compared with corresponding normal tissue counterparts, several cancer types including breast have significantly elevated LSD2 mRNA expression (Figure 1C, Supplementary Table 1) (TCGA PANCAN RSEM TPM data downloaded from https://toil.xenahubs.net). Overexpression of LSD2 in several pathological types of breast cancer was also found in METABRIC dataset (Curtis Breast) (Supplementary Table 2) (https://www.oncomine.org). Further analysis of LSD2 expression across all molecular subtypes of breast cancer showed that LSD2 mRNA level is significantly higher in basal-like tumors as compared to other breast cancer subtypes or normal tissues (Figure 1D) (TCGA data downloaded from GSE62944). Taken together, these data suggest a consistent increase of LSD2 expression in breast cancer cell lines and clinical tumor samples warranting further investigation into the role of LSD2 in breast cancer progression.

### LSD2 promotes breast cancer cell growth and colony formation

**Figure 2:**
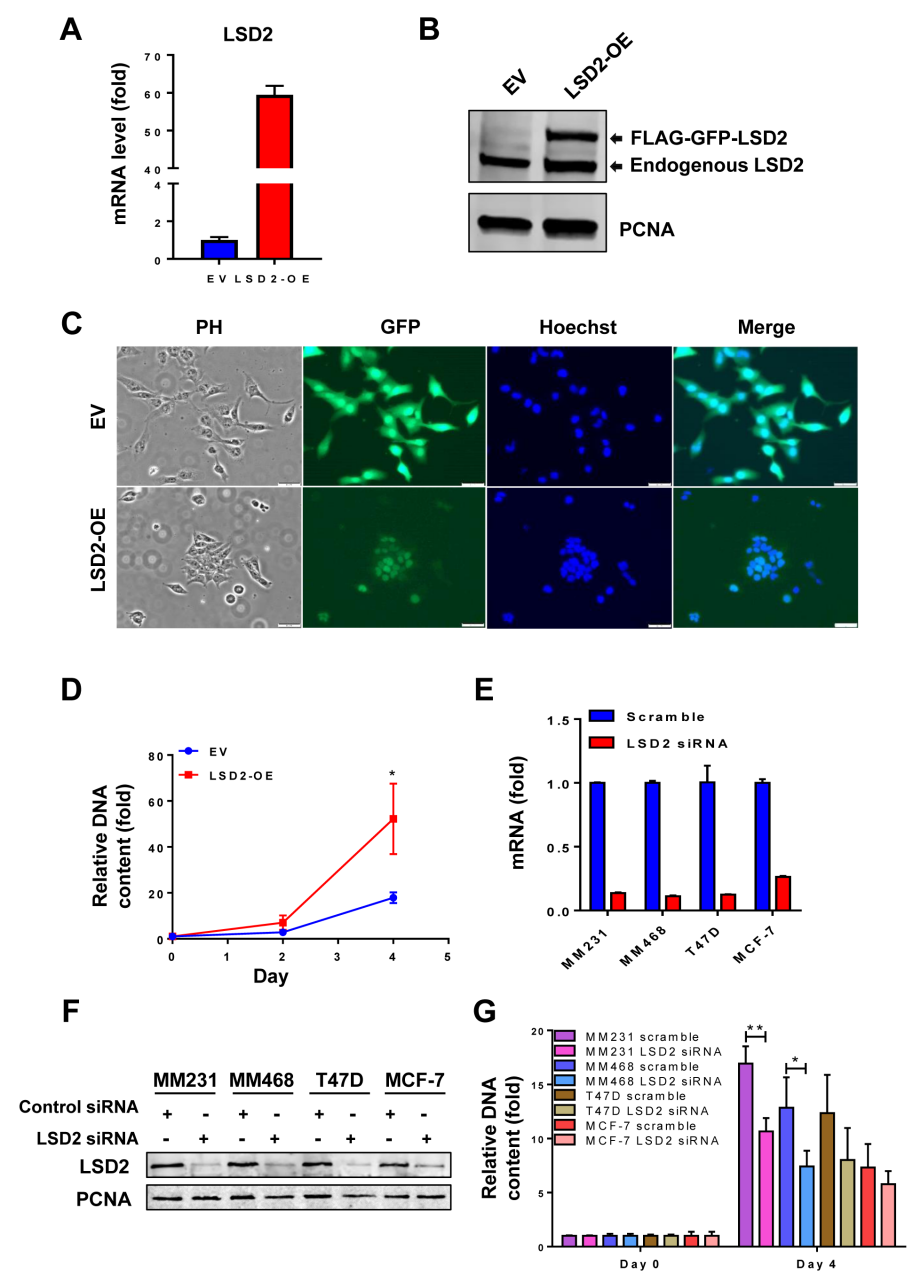
Effect of LSD2 overexpression or depletion on proliferation of breast cancer cells **A.** MDA-MB-231 cells were transfected with control empty vector (EV) or LSD2 overexpression vector (OE) for 48 h followed by selection with G418. mRNA expression of LSD2 was measured by quantitative real-time PCR with GAPDH as an internal control. **B.** Cellular nuclear proteins were extracted, and LSD2 protein expression in MDA-MB-231-EV or LSD2-OE cells was examined by Western blots using anti-LSD2 antibody with proliferating cell nuclear antigen (PCNA) as an internal control. **C.** MDA-MB-231 cells transfected with control empty vector (EV) or LSD2 overexpression vector (LSD2-OE) were fixed with 4% PFA followed by Hoechst 33258 staining. Bright field and fluorescent images were taken to observe cellular morphology and LSD2-GFP protein expression. PH, Phase Contrast. **D.** MDA-MB-231 cells transfected with control empty vector (EV) or LSD2 overexpression vector (LSD2-OE) were analyzed for growth using fluorometric dsDNA quantitation method. **E.** Human breast cancer MDA-MB-231, MDA-MB-468, MCF-7 and T47D cells were transfected with scramble or LSD2 siRNA for 96 h followed by qPCR examination of LSD2 mRNA expression level. β-actin was used as an internal control. **F.** Cells transfected with scramble or LSD2 siRNA were examined for LSD2 protein expression by western blots with PCNA as an internal control. **G.** Fluorometric dsDNA quantitation assays were performed to evaluate growth of breast cancer cells which were transfected with scramble or LSD2 siRNA for 96 h. All experiments were performed at least three times and bars represent the means of three independent experiments ± s.d. * *p* < 0.05, ** *p* < 0.01, Student’s t-test.

To explore the functional role of LSD2 in regulating breast cancer development, we stably overexpressed eGFP and Flag-dually tagged LSD2 in MDA-MB-231 (LSD2-OE) and validated the overexpression at the mRNA and protein levels (Figure 2A and 2B). Tracking of the GFP tag through fluorescent microscopy showed that the LSD2-eGFP-Flag localizes exclusively to the nucleus in MDA-MB-231 cells (Figure 2C). While cells transfected with control empty vector (EV) display the spindle shaped morphology of parental MDA-MB-231 cells, LSD2 overexpression induces a cobblestone-like morphology with apparent cell-cell adhesion (Figure 2C).

Next, we investigated the potential impact of increased LSD2 expression on breast cancer cell proliferation. Cellular proliferation assays showed that stable overexpression of LSD2 in MDA-MB-231 cells significantly promoted cellular growth rate (Figure 2D). To further validate this phenotypic change, two basal-like/triple-negative breast cancer (TNBC) cell lines, MDA-MB-231 and MDA-MB-468, and two luminal/Estrogen Receptor positive (ER+) cell lines, T47D and MCF-7, were transfected with non-targeting scramble or LSD2-specific siRNA. LSD2-targeting siRNA effectively suppressed endogenous LSD2 mRNA and protein expression in all lines (Figure 2E and 2F). Although depletion of LSD2 hindered the cell proliferation in all lines, this effect was more pronounced and statistically significant in TNBC cell lines as compared to ER+ cell lines (Figure 2G).

**Figure 3:**
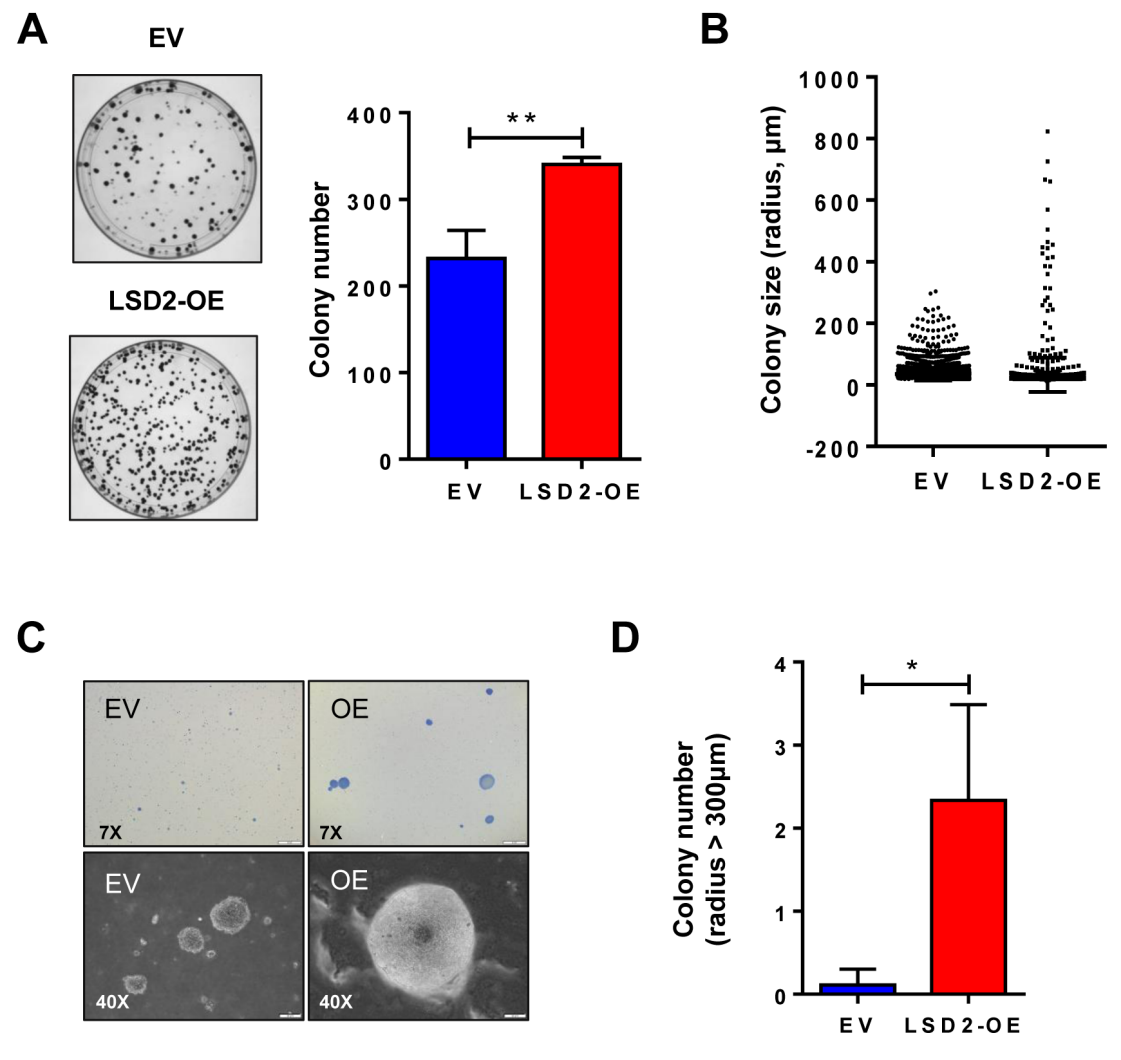
LSD2 enhances the colony formation capacity of MDA-MB-231 cells **A.** 500 cells stably transfected with empty vector or LSD2 expression plasmids were plated in 10cm dish. After 14 days, colonies formed were stained with 0.5% crystal violet and counted. **B.** 10,000 cells per dish were seeded in 0.4% soft agar in 35mm dish. After 3 weeks, colonies were stained with 0.005% crystal violet and counted using CellSens software. Individual colonies formed by empty vector control or LSD2 overexpressing MDA-MB-231 cells were plotted based to colony size (μm). **C.** Representative microscopy images (7× and 40×) of cellular colonies after 3 weeks of seeding the cells on soft agar coated wells. **D.** Average numbers of colony whose radius is over 300 mm. Error bar represents ± s.d. from three independent experiments. * *p* < 0.05, ** *p* < 0.01, Student’s t-test.

Our previous study demonstrated that shRNA-mediated inhibition of LSD2 leads to a significant reduction in 2D colony formation in MDA-MB-231 cells, indicating a survival-promoting role for LSD2 in breast cancer cells [21]. In this study, we investigated the effect of LSD2 overexpression on 2D colony formation of MDA-MB-231 cells. In agreement with the effect of LSD2 knockdown, ectopic expression of LSD2 in MDA-MB-231 cells significantly increases the number of 2D colonies (Figure 3A). We then extended our investigation to an anchorage-independent soft-agar colony formation assay to further dissect the role of LSD2 in breast tumorigenicity. The soft agar results showed that, although there was no significant difference in average colony size (Figure 3B), LSD2-OE cells developed an increased number of larger colonies (> 300 μm) than empty vector cells (Figures 3B, 3C and 3D). Collectively, these results suggest that LSD2 enhances *in vitro* colony formation capacity of breast tumor cells.

### LSD2 attenuates motility and invasion of breast cancer cells

**Figure 4:**
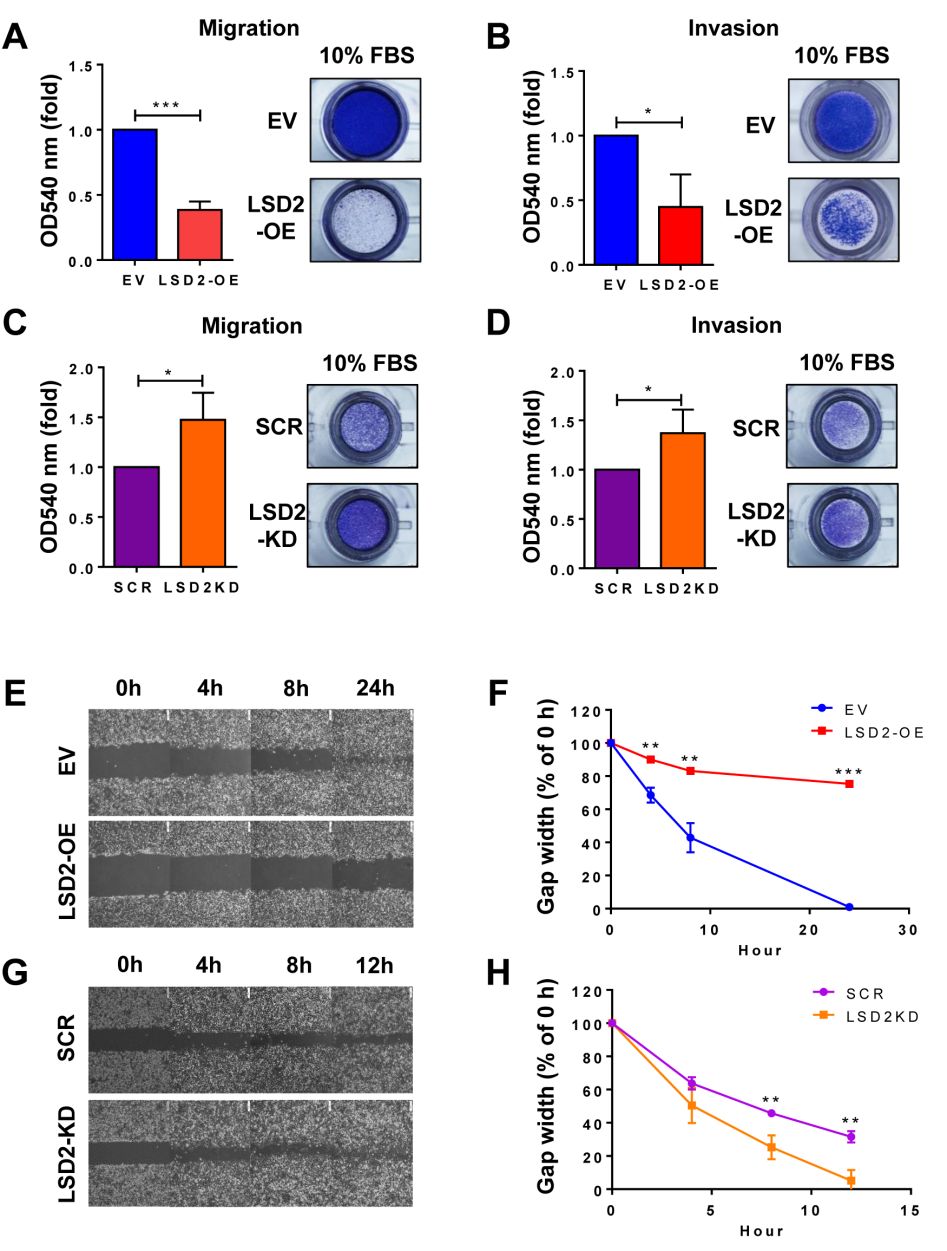
LSD2 regulates migration and invasion in MDA-MB-231 cells **A.** Transwell migration assay was performed to detect the migratory capacity of MDA-MB-231 EV and LSD2-OE cells. Quantification of the migrated cells was done by solubilization of crystal violet and spectrophotometric reading at OD 540. **B.** Quantification of the invasive MDA-MB-231 EV and LSD2-OE cells. Transwell invasion assay was performed and the invasive cells were quantified by solubilization of crystal violet and spectrophotometric reading at OD 540. **C.** Quantification of the migratory MDA-MB-231 cells transfected with scramble and LSD2 shRNA plasmids. **D.** Quantification of the invasive MDA-MB-231 cells transfected with scramble and LSD2 shRNA plasmids. **E.** Confluent monolayers of EV and LSD2-OE MDA-MB-231 cells were wounded by scratch with a pipette tip. Cells were then incubated for 24 h. Images were taken at the end points to be compared to 0 h to measure wound healing. **F.** The average of wound closure rate during the first 24 h of wound healing was calculated. **G.** Confluent monolayers of scramble shRNA and LSD2-KD MDA-MB-231 cells were wounded by scratch. Cells were then incubated for 12h. Images were taken at the end points to be compared to 0 h to measure wound healing. **H.** The average of wound closure rate during the first 12 h of wound healing was measured and quantified. All experiments were independently performed at least three times and values represent the mean ± s.d. * *p* < 0.05, ** *p* < 0.01, *** *p* < 0.001, Student’s t-test.

Enhanced motility and invasion are positively associated with the aggressive behavior and poor prognosis of breast cancer. We anticipated that accelerated growth rate by LSD2 overexpression would lead to corresponding augmentation of cellular motility and invasion and tested this hypothesis through transwell Boyden chamber assays. Unexpectedly, we found that LSD2 overexpression significantly reduced migration and invasion of MDA-MB-231 cells (Figure 4A and 4B). To validate this result, we performed the same experiments using a pool of MDA-MB-231 cells stably expressing shRNA against LSD2, which decreased LSD2 mRNA expression by about 75% as compared with scramble control cells (Supplementary Figure 1). Boyden chamber assays demonstrated that loss of LSD2 facilitated cell migration and invasion of MDA-MB-231 cells (Figure 4C and 4D). To further verify these results, we performed *in vitro* wound-healing assay and found that MDA-MB-231 cells transfected with control empty vector closed the wound much more efficiently than LSD2-overexpressing cells (Figure 4E and 4F). On the contrary, inhibition of LSD2 in MDA-MB-231 cells significantly augmented the wound-healing rate (Figure 4G and 4H). Collectively, these results point to an inhibitory role of LSD2 in mediating breast cancer cell migration and invasion.

### LSD2 overexpression promotes breast cancer stem cell-like characteristics

**Figure 5:**
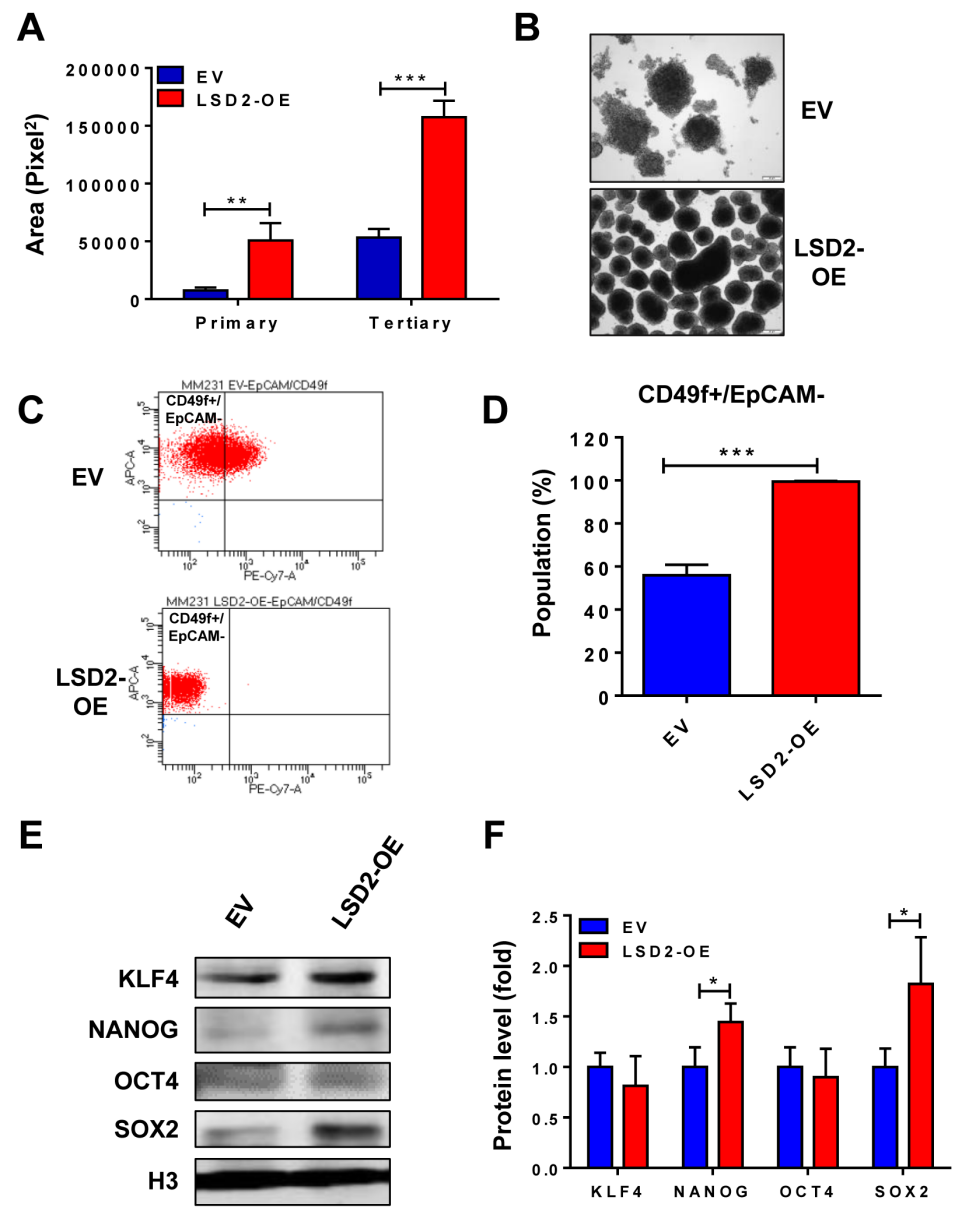
Overexpression of LSD2 facilitates breast cancer stem cell characteristics **A.** MDA-MB-231 EV or LSD2-OE cells were suspended in tumor sphere medium and seeded in 6-well plate with ultra-low attachment surface. After 7-day incubation, spheres were collected and digested into single cells. Same density of digested cells was seeded for secondary mammosphere and tertiary mammosphere formation. Quantification of primary and tertiary mammospheres was performed using CellSens software. **B.** Representative pictures of tertiary mammospheres formed by EV and LSD2-OE cells. **C.** Flow cytometry analysis of cell surface marker CD49f and EpCAM in EV and LSD2-OE cells. **D.** The percentage of CD49f^+^/EpCAM^-^ cells was quantified from three independent experiments. **E.** Western blot examinations on nuclear protein levels of KLF4, NANOG, OCT4 and SOX2 in EV and LSD2-OE cells. Histone 3 (H3) was used as internal control. **F**. The experiments were performed three times with similar results. Values represent means ± s.d. * *p* < 0.05, *** *p* < 0.001, Student’s t-test.

Breast cancer stem-like cells (BCSCs) possess features of multipotent, oncogenic, and self-renewal capacity, which are responsible for breast tumor heterogeneity [22, 23]. Recent studies have shown that LSD1 plays a critical role in promoting the differentiation and self-renewal of cancer stem cells (CSCs) in human breast cancer and in other cancer types [24, 25]. To elucidate the potential implication of LSD2 in breast cancer stem cell phenotypes, mammosphere formation assay was carried out, which showed that LSD2 overexpression significantly increases the size and number of both primary and tertiary spheres (Figure 5A and 5B), suggesting the enrichment of a subpopulation of CSCs with self-renewal capacity in LSD2-OE cells. Flow cytometry analysis of LSD2-OE cells indicated a significantly increased CD49f+/EpCAM- subpopulation, which is considered to be enriched for stem/basal progenitor cells (Figure 5C and 5D). We also examined the nuclear protein expression of four embryonic stem cell (ESC) markers, KLF4, NANOG, OCT4 and SOX2 and observed that LSD2 overexpression increases expression of NANOG and SOX2 (Figure 5E and 5F). Finally, we investigated the level and activity of Aldehyde Dehydrogenase (ALDH) in LSD2-OE cells. Recent studies indicate that enhanced ALDH activity is a hallmark of cancer stem cells [26, 27]. In line with previous report that MDA-MB-231 cells express very low level of ALDH (0%-1% positive) [28], no obvious ALDH^high^ cells were detected in MDA-MB-231 EV cells (around 0%) whereas LSD2 overexpression increased ALDH^high^ cell population to about 1.5% (Supplementary Figure 2). In addition, mRNA expression of many ALDH family members was increased by LSD2-OE based on our recently microarray study (Supplementary Table 3). Collectively, all these data point to the critical function of LSD2 in promoting BCSC-like properties.

### Overexpression of LSD2 alters expression of key epigenetic modifiers

**Figure 6:**
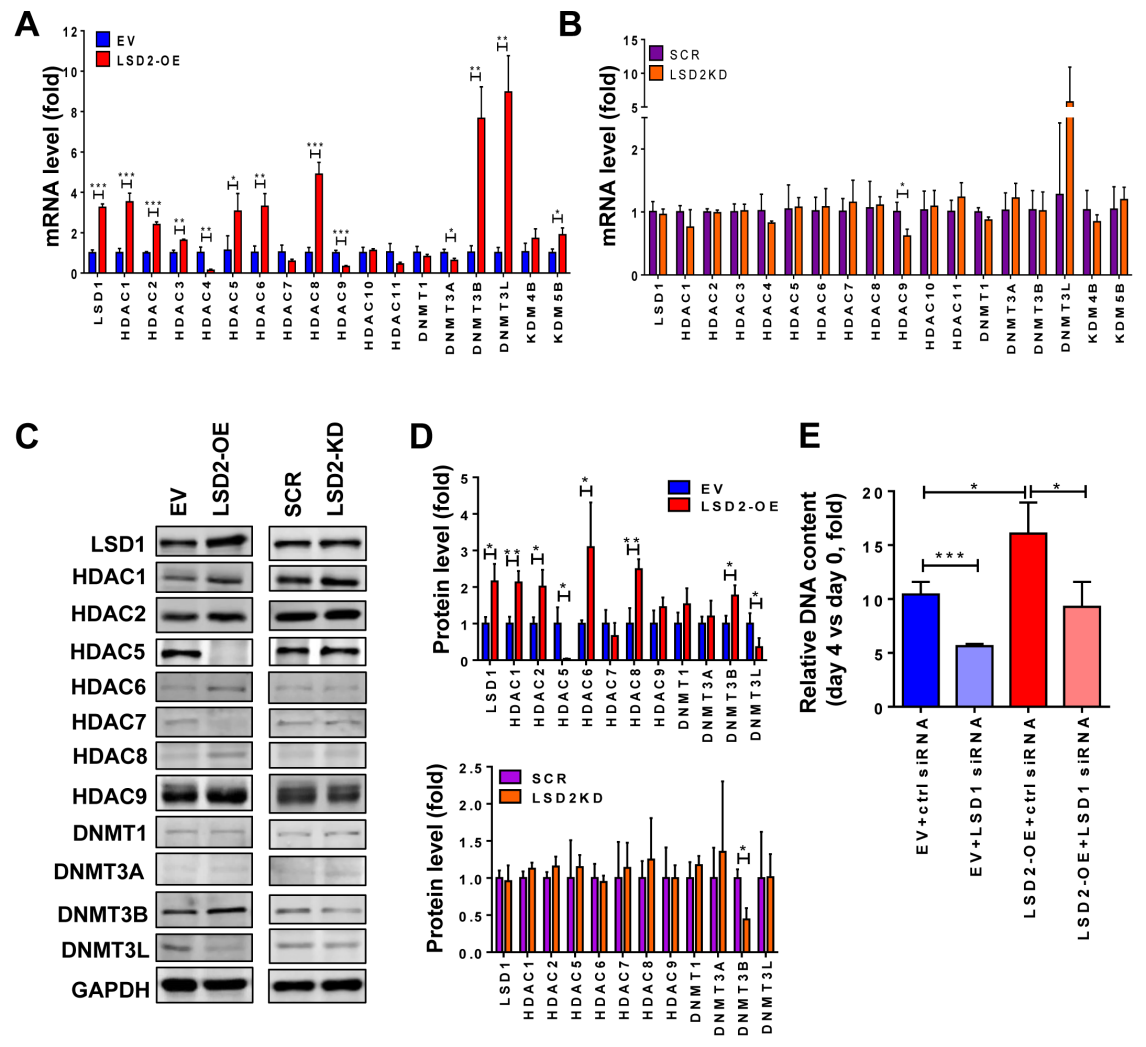
Effect of LSD2 on expression of key epigenetic modifiers **A.** RNA was extracted from MDA-MB-231 EV and LSD2-OE cells and cDNA was synthesized and subjected to quantitative real-time PCR for the indicated genes using TaqMan probes. GAPDH expression was used as an internal standard. **B.** mRNA expression of chromatin modifying factors in MDA-MB-231 cells stably transfected with scramble (SCR) or LSD2 shRNA (LSD2-KD). GAPDH expression was used as an internal standard. **C.** Indicated chromatin modifying factors were analyzed for their protein levels by western blots in MDA-MB-231 EV, LSD2-OE, scramble shRNA and LSD2-KD cells. GAPDH was used as a loading control. **D.** Histograms represent the average protein levels of indicated chromatin modifiers in three independent experiments relative to GAPDH protein ± s.d. as determined by quantitative immunoblots. **E.** MDA-MB-231 EV and LSD2-OE cells were transfected with scramble or LSD1 targeting siRNA for 96 h followed by growth assay using fluorometric dsDNA quantitation. Column with error bar represents mean ± s.d. from three independent experiments. * *p* < 0.05, ** *p* < 0.01, *** *p* < 0.001, Student’s t-test.

Our recent studies have revealed that dysregulated regulatory networks formed by aberrant crosstalk between histone methylation and histone acetylation or DNA methylation profoundly impact breast cancer progression [13, 15, 21, 29]. To explore the involvement of LSD2 in these regulatory processes, we assessed the impact of LSD2 overexpression or deficiency on mRNA and protein expression of key members of DNMT, HDAC and KDM families. Quantitative RT-PCR results showed that LSD2 overexpression significantly increased the mRNA levels of LSD1, HDAC1, 2, 3, 5, 6, 8, DNMT3B and 3L, KDM4B and KDM5B (Figure 6A). On the other hand, expression of only a few genes was affected by LSD2 stable knockdown, including HDAC9 and DNMT3L (Figure 6B). In LSD2 siRNA-transfected MDA-MB-231 cells, mRNA levels of LSD1, HDAC4, and DNMT3B were decreased while HDAC1 mRNA level was increased (Supplementary Figure 3). The protein expression of several genes was further tested to determine if there is correlated alteration between mRNA and protein expression. Quantitative western blots showed that LSD2 overexpression significantly increased the protein expression of LSD1, HDAC1, 2, 6, 8 and DNMT3B, and inhibited the expression of HDAC5 and DNMT3L (Figure 6C), whereas DNMT3B was the only factor altered by LSD2-KD (Figure 6D).

Increased LSD1 expression in LSD2-OE cells raises an important question as to whether the tumor growth promoting activities of LSD1 and LSD2 are interdependent. To address this question, a rescue experiment was carried out to knock down LSD1 expression by siRNA in EV and LSD2-OE cells. Treatment with siRNA effectively depleted the mRNA expression of LSD1 without altering LSD2 expression levels (Supplementary Figure 4). Rescue with LSD1 siRNA hindered the growth of both MDA-MB-231 EV and LSD2-OE cells, but exhibited a similar extent of rescue efficiency (decreases of about 35% vs 39%) (Figure 6E). This result clearly indicates that LSD2 promotes breast cancer cell proliferation in an LSD1-independent manner.

### Overexpression of LSD2 promotes growth and inhibits lung metastasis of MDA-MB-231 xenograft tumors in nude mice

**Figure 7:**
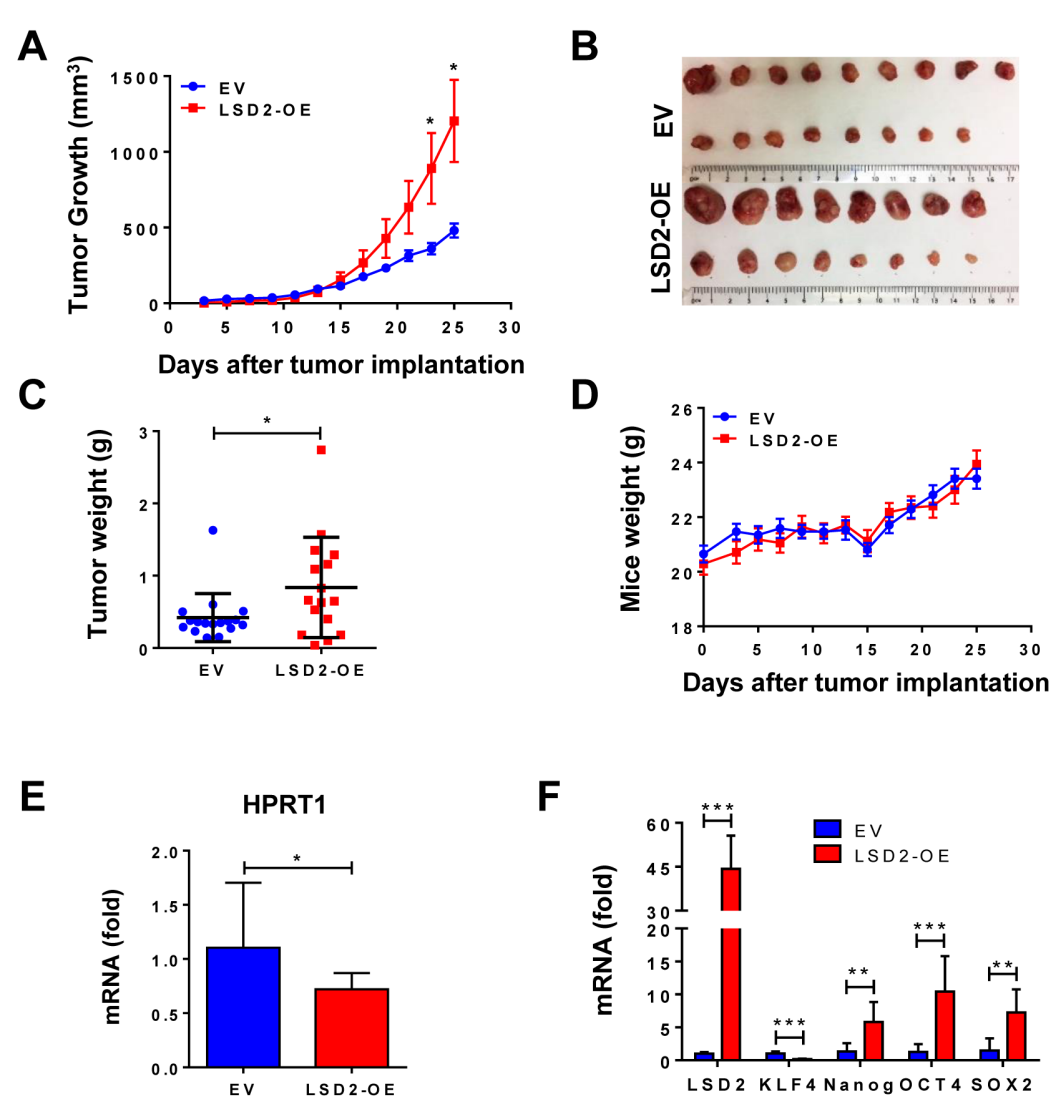
*In vivo* effect of LSD2 on proliferation and metastasis in mice bearing MDA-MB-231 xenograft **A.** MDA-MB-231 cells transfected with empty vectors (*n* = 17) or LSD2 expression vectors (*n* = 16) were transplanted into the mammary fat pad of nude mice. Tumor volumes were regularly assessed every two days. Shown are average tumor volumes ± s.e. **B.** Orthotopically implanted tumors were removed after terminating the experiments. Shown are pictures of implanted tumors. **C.** Weight of individual animal tumor was measured at the end of experiment. **D.** Weights of mice were measured on the indicated days. Points, mean mouse weight (g); bars, mean ± s.d. **E.** Tumor cells metastasized to mice lung were assessed by quantification of mRNA expression of human HPRT1 gene (EV, *n* = 10; LSD2-OE, *n* = 16). Mouse b-actin was used as internal control. Graph was plotted as fold change with normalization to EV. **F.** Total RNA was extracted from 7 randomly selected tumors from each group and mRNA levels of the four embryonic stem cell markers were evaluated by qPCR. *p < 0.05, **p <0.01, ***p < 0.001, Student’s t-test.

To confirm our *in vitro* results, we implanted MDA-MB-231 EV and LSD2-OE cells into the mammary fat pads of athymic nude mice. LSD2 overexpression led to accelerated tumor growth, with approximately three-fold increase in average tumor size over empty vector cells (Figure 7A and 7B). Statistical analysis of *in vivo* tumor growth is summarized in Supplementary Table 4. Average weight of LSD2-OE tumors was statistically higher than control group at the end of the experiment (Figure 7C). Both groups of animals had normal body weight gains (Figure 7D). To evaluate *in vivo* effect of LSD2 on tumor metastasis, we quantified mRNA expression of human housekeeping gene HPRT1 in mouse lung tissue samples by real-time RT-PCR using a probe that does not cross-react with its mouse counterpart. Our results showed that mRNA level of hHPRT1 gene was significantly reduced in lung tissues of mice bearing LSD2-OE tumors (Figure 7E). Normal mouse lung tissue was used as a negative control, and no expression of hHPRT1 was detected, thus validating the specificity of the hHPRT1 probe (Data not shown). To determine the *in vivo* impact of LSD2 overexpression on cancer stem cell markers, qPCR analysis was performed on RNA from tumors, which showed that the mRNA expression of NANOG, OCT4 and SOX2 were significantly induced in LSD2-OE xenograft tumor cells (Figure 7F). In agreement with *in vitro* results, the findings from this mouse study suggest that LSD2 promotes breast tumor growth and BCSC characteristics, while simultaneously attenuating cell invasion and dissemination *in vivo*.

## DISCUSSION

Histone demethylases have emerged as a novel class of epigenetic regulators controlling cancer initiation and progression [30]. Dysregulated expression and functions of histone lysine demethylases are found in many types of cancers, and thus represent novel promising therapeutic targets for cancer. In the past decade, rapid progress has been made in understanding the molecular basis of histone demethylase-dependent functions in breast cancer biology [16, 20]. Among these enzymes, LSD1 is the first recognized histone lysine demethylase and perhaps one of the best-characterized histone-targeted enzymes in breast cancer. However, the involvement of LSD2, the only identified homolog of LSD1, in breast cancer is still very elusive. *In silico* data indicate a significant elevation of LSD2 expression in aggressive basal-like breast tumors as compared with other breast cancer subtypes and normal tissues, suggesting a potential link between LSD2 overexpression and aggressiveness of breast cancer. However, the molecular mechanism of LSD2 upregulation in breast cancer and the long-term clinical impact of elevated LSD2 expression in the risk stratification of breast cancer patients are still unclear. Therefore, more robust studies are needed to clarify these questions.

While LSD1 is typically associated with oncogenic phenotypes in almost all types of cancer, little is known about the function of LSD2 in mediating tumor progression. A recent study by Yang *et al* reported that LSD2 acts as an E3 ubiquitin ligase and inhibits A549 lung cancer cell growth through proteasomal degradation of O-GlcNAc transferase (OGT) [31], suggesting that LSD2 may inhibit the growth of certain types of cancer in a ubiquitination-dependent manner. The *in vivo* effect of LSD2 on A549 cell growth warrants further examination. In our study, we utilized both *in vitro* and *in vivo* models to investigate the potential implication of LSD2 in regulating breast cancer proliferation and metastasis. We found that overexpression of LSD2 in breast cancer cells consistently enhances MDA-MB-231 cell growth *in vitro* as well as in tumor xenografts in mice, whereas depletion of LSD2 by siRNA hinders the growth of multiple breast cancer cell lines. We also showed that LSD2 overexpression increases the number of colonies in 2D monolayer culture and large colonies in anchorage-independent 3D culture, indicating that LSD2 may potentiate the malignant transformative capacity of breast cancer cells. Interestingly, overexpression of LSD2 results in an increase of mRNA and protein expression of LSD1. A rescue study demonstrated that simultaneous treatment with LSD1 siRNA in control and LSD2-OE cells exerts similar effect on LSD2-mediated tumor cell growth. This result suggests that LSD1 and LSD2 may have non-redundant roles in promoting breast cancer proliferation.

The concept of breast cancer stem cells (BCSCs) was first introduced by Al-Hajj *et al* [32]. BCSCs are a rare subpopulation that originates from a small fraction of tumor initiating cells with the abilities of self-renewal, unlimited propagation and multipotent differentiation. Importantly, BCSCs are associated with poorer clinical outcome and are intrinsically resistant to therapy. Wu *et al* recently reported that the deubiquitinase USP28 promotes breast cancer stem cell (BCSC)-like characteristics *in vitro* and *in vivo* through stabilizing LSD1 protein [24]. We explored the potential regulation of LSD2 on BCSC features and showed that LSD2 overexpression facilitates the formation of several generations of mammospheres, enriches the CD49f^+^/EpCAM^-^ stem/basal progenitor subpopulation and promotes the expression of several pluripotent stem cell markers *in vitro* and in MDA-MB-231 xenograft tumors. Our findings indicate that, like LSD1, LSD2 has an important role in conferring CSC-like traits to breast cancer cells. In ESCs, the histone modification landscape profoundly influences the crosstalk of transcriptional regulators [33, 34]. Increasing lines of evidence suggest that the two key histone marks, H3K4 methylation and H3K27 methylation, serve as critical histone bivalent marks controlling developmental regulatory genes in embryos and ESCs [33, 35, 36]. LSD1 has been shown to act as a key histone modifier in the maintenance of pluripotency by occupying the promoter of a subset of developmental genes containing bivalent domains (H3K4 di/trimethylation and H3K27 trimethylation marks) and regulating the balance between self-renewal and differentiation in human ESCs [37]. It is probable that LSD2, in collaboration with LSD1, provides an additional layer of epigenetic modification in governing breast cancer stem cell features through modulation of the level of H3K4 methylation at pluripotent regulatory genes. Future study using genome-wide mapping approaches would aid in probing the subset of LSD2 target genes and histone mark alterations that are associated with biological processes in BCSC development.

Our studies point to potentially opposite roles of LSD2 in regulating breast cancer cell growth and invasion. Our *in vivo* study validated *in vitro* results showing that lung metastasis is attenuated in mice bearing LSD2-overexpressing tumors. This opposite effect may reflect a broad and complex involvement of LSD2 in regulating histone function and gene transcriptional activities that could ultimately up-regulate growth-associated gene expression, while suppressing motility and invasion genes. Indeed, several other studies have reported that a number of genes possess opposite effects on cancer proliferation and metastasis [38, 39]. Morphologically, MDA-MB-231 LSD2-OE cells acquire tightly cohesive, cobblestone-like epithelial cell morphology as compared to the elongated fibroblast-like control cells. This finding suggests that increased LSD2 expression may induce a mesenchymal-epithelial transition (MET) through acquisition of epithelial markers with concurrent loss of mesenchymal features, which in turn leads to loss of migratory and invasive ability of tumor cells. Indeed, a number of genes involved in tight junction or apical-basal polarity such as OCLN, DSP, SCRIB, etc., were upregulated by LSD2-OE while VIM and FN1 were downregulated according to results of our recent microarray analysis (Supplementary Table 5). Some early studies have revealed that activated EMT program in non-transformed epithelial cells could confer properties of stem cells which may facilitate the development of tumor initiating cells [40]. However, a number of groups have recently reported that EMT may not be necessarily associated with cancer stemness features. For example, Schmidt *et al.*, have shown that activities of EMT and stemness are somehow antagonistic and attenuation of the EMT process is required for the full acquisition of stem cell properties [41]. The Weinberg lab demonstrated that the EMT program may not be sufficient to induce changes of stemness in differentiated luminal cells, and additional genetic programs are needed to interact with EMT environment to induce phenotypic alteration of cancer stemness [42]. Future studies using appropriate *in vitro* and *in vivo* models are required to completely understand the precise role of LSD2 in regulating cross-talk between EMT/MET and stemness and its relevance in breast cancer progression and metastasis.

Our study also revealed that the expression levels of many key chromatin modifiers are altered by LSD2 overexpression, indicating a significant role of LSD2 in the epigenetic regulatory network in breast cancer cells. For example, stable LSD2 overexpression significantly increases the expression of LSD1, HDAC1, and HDAC2, which are important components of the NuRD (nucleosome remodeling and histone deacetylase) complex that has important implications in cancer biology [43, 44]. LSD2 overexpression also promotes the expression of DNMT3B, which is a critical epigenetic player in inducing aberrant DNA methylation and gene silencing in cancer [45]. The molecular mechanisms linking LSD2 to transcriptional regulation remain elusive. A study by Fang *et al* used ChIP-chip tiling array to map LSD2 binding loci on a genome-wide scale and found that, in addition to H3K4 demethylase activity, LSD2 may act as a positive regulator of gene transcription through binding to highly transcribed coding regions enriched in active histone marks such as H3K36me3 [6]. They also reported that LSD2 forms a complex with euchromatic histone methyltransferases EHMT1/2 and NSD3 as well as active transcription elongation factors such as Pol II and cyclin T1 [6]. We also noted that stable and transient knockdown of LSD2 exerted distinct impact on expression of epigenetic modifiers. It is possible that long-term suppression of LSD2 may intrinsically alter the genomic expression of other proteins and leads cells to compensate by increasing or reducing the expression of other signaling proteins. Further investigation is required to define the exact mechanisms by which LSD2 alters transcription of key epigenetic modifiers through mediating histone disassembly/reassembly and transcription elongation at gene coding regions.

In summary, our studies provide novel insight into the previously unrecognized roles of LSD2 in human breast cancer cells. We have shown for the first time that LSD2 augments proliferative and cancer stem cell traits, and attenuates motility and invasiveness of breast cancer cells. All of these findings suggest that LSD2 has complex and multifaceted roles in breast oncogenesis. In the future, better understanding of epigenetic downstream target genes and pathways controlled by LSD2 would aid in developing novel small molecule inhibitors and combination strategies which might confer selective effects against breast cancer.

## MATERIALS AND METHODS

### Cell lines and culture conditions

Human breast cancer cell lines MDA-MB-231, MDA-MB-468, MCF-7, T47D and normal immortalized breast epithelial cell line, MCF10A, were obtained from the ATCC/NCI Breast Cancer SPORE program. Cells were cultured in growth medium as described previously [15, 46]. Stable transfectant lines were maintained with 800 µg/mL G418 (Geneticin).

### Plasmid construction and stable transfection

Full length human LSD2 cDNA from MCF-7 cells was originally cloned by PCR into pcDNA3.1/V5-His TOPO. PCR primers engineered with KpnI sites were used to amplify LSD2 and then cloned into eGFP-Flag vector (using KpnI site in MC1) purchased from Gene Copoeia (Rockville, MD). Empty eGFP-flag vector (EV) or LSD2-eGFP-Flag (LSD2-OE) was transfected into MDA-MB-231 cells using Lipofectamine 3000 (Thermo Fisher Scientific, Waltham, MA) according to manufacturer’s instructions. After 48-hour transfection, cells were selected with 800 µg/mL G418 for several weeks. Then eGFP-positive cells were further sorted three times by flow cytometry to enrich LSD2-eGFP-Flag overexpressing cells.

### Small interfering RNA treatment

Pre-designed LSD2 or LSD1 siRNA and non-targeting scramble siRNA (Santa Cruz Biotechnology, Dallas, TX) were transfected into cells following manufacturer’s protocol. Briefly, cells were seeded in 96-well plates the day before transfection. siRNA was prepared in transfection medium (sc-36868) with transfection reagent (sc-29528). Cells were washed using transfection medium before 100 µL of siRNA complexes were added. After 5-hour incubation at 37°C, 100 µL normal growth medium containing 2× fetal bovine serum was added to each well. After 96-hour incubation, relative cell number was evaluated using FluoReporter Blue Fluorometric dsDNA Quantitation Kit (Thermo Fisher Scientific) according to manufacturer’s protocol.

### shRNA treatment and stable cell line generation

Scramble and 4 different LSD2 shRNAs were purchased from SABiosciences (Germantown, MD) and reverse transfected with Attractene transfection reagent (using GFP expression plasmids first, followed by Gentamycin expression plasmids) into MDA-MB-231 cells. At 48 h post-transfection, cells were first selected with 800 μg/ml G418 for several weeks, and then sorted by flow cytometry to enrich for GFP+ cells. All transfections were assayed by qPCR and western blot analysis for the best knockdown efficiency.

### RNA extraction and qPCR

Total RNA was extracted using RNeasy kit (Qiagen, Valencia, CA) following manufacturer’s instructions. Tissues were directly homogenized in RNA lysis buffer which in this kit is RLT buffer. cDNA was synthesized using M-MLV Reverse Transcriptase (Thermo Fisher Scientific). Quantitative real-time PCR was performed on the StepOne real-time PCR system using TaqMan Gene Expression Assays (ThermoFisher Scientific).

### Immunoblotting

Whole cell lysate and nuclear proteins were extracted as described previously [15, 21, 29]. Briefly, 60 μg whole cellular protein or 30 μg nuclear protein was separated on Mini-PROTEAN^®^ TGX™ 4-20% acrylamide gels and transferred onto NC membranes. Antibodies used in this study are shown in Supplementary Table 6. CD49f-APC and EpCAM-PE-Cy7 antibodies (BD Biosciences, Franklin Lakes, NJ) were provided by Dr. Mei Zhang (University of Pittsburgh Cancer Institute). Membranes were scanned with Odyssey Infrared Imaging System (Li-Cor Biosciences, Lincoln, NE).

### Cell proliferation assay

Cells were seeded at 1000 to 5000 cells per well in 96-well plates. At each time point, medium was discarded by inverting the plates. Then the plates were frozen in -80°C freezer until ready to be measured. 100 μl distilled water was added into each well after the plates were thawed to room temperature. Then the plates with water were incubated at 37°C for 1h. Plates were frozen and thawed again to lyse the cells in order to release DNA completely. The DNA content was measured using FluoReporter Blue Fluorometric dsDNA Quantitation Kit (Thermo Fisher Scientific) by adding 100 µL of aqueous Hoechst 33258 in TNE buffer into each well and then measured using VICTOR X4 plate reader (PerkinElmer, Waltham, MA).

### Monolayer culture colony formation assay

Empty vector and LSD2-OE MM231 cells were seeded at 500 cells per 10cm dish. After 14 days, cells were stained with 0.5% crystal violet, dried overnight and colonies were counted. Colonies that contained >50 cells were scored. All experiments were carried out independently at least three times. The results were expressed as means ± s.d.

### Soft agar colony formation assay

1.2% Bacto-agar (BD Biosciences) was autoclaved and then warmed to 42°C. By mixing 1.2% agar with growth medium 1:1, 0.6% agar/medium was generated and then 1.5 ml of the mixture was quickly plated into 35mm dishes as base layer. Solidification was completed at room temperature for 45 min. Then 4.5×10^4^ cells were suspended in 3 ml growth medium supplemented with 3× serum and non-essential amino acids (NEAA, Thermo Fisher), then mixed with 1.5 ml 1.2% agar. The resulting mixture, 1 ml of cells/0.4% agar/medium (10,000 cells/ml) was quickly and gently added onto each plate for solidification. Formed colonies were examined using SZX-16 microscope and analyzed by CellSens Dimension software (Olympus, Shinjuku, Tokyo, Japan).

### Transwell cell migration and invasion assays

Cells were starved in serum-free DMEM for 24h before the experiment. Then cells were harvested, washed and counted. Appropriate amounts of pre-warmed medium (no serum or 10% FBS) was added to the wells, then the inserts were carefully put into these wells using sterile forceps (for migration assays, we used Corning 8.0um PET track-etched membrane, 24 or 12 well format; for invasion assays, we used Corning Biocoat Matrigel Invasion Chamber, 24 well format). Then 1×10^5^ cells (for 24 well plates) or 5×10^5^ cells (for 12 well plates) in serum-free DMEM were added to the inserts. After 48h incubation, cells migrated through the membrane were stained with 0.5% crystal violet and cells not migrated through were removed using cotton swab. The stain was dissolved in 0.1M Sodium Citrate and the absorbance was read at 540nm on a plate reader.

### Scratch wound healing assay

1×10^6^ cells per well were placed in a 6-well plate. The “wound” was made by scratching the confluent monolayer across the well using a 200 μl pipette tip. At each time point, closure of the gap was recorded by taking pictures. Then the width of the gap was measured and normalized with 0 h.

### Mammosphere formation assay

The mammosphere assay was developed as an approach to propagate mammary epithelial stem cells [47]. This assay was performed according to an online protocol (http://www.bio-protocol.org/e325). Briefly, tumorsphere medium was made by adding 20ng/ml epidermal growth factor (EGF), 10ng/ml basic fibroblast growth factor (bFGF), 5ug/ml Insulin and 0.4% Bovine Serum Albumin in DMEM/F12 (50/50) medium, and B27 supplement (Thermo Fisher) was freshly added to tumorsphere medium. Cells were collected, washed and counted followed by resuspending in tumorsphere medium with B27 supplement at a final concentration of 10,000 cells/ml. Then 2 ml cells were added to each well of an ultra-low attachment 6-well plate (Corning). After 7-day incubation, pictures of each well were taken and colonies were quantified using CellSens Dimension software. Secondary or tertiary mammospheres were generated by digesting primary mammospheres or secondary mammospheres and were seeded at the same density as primary mammospheres. All experiments were performed three times and bars represent the means of three independent experiments ± s.d.

### Flow cytometry analysis

1×10^6^ cells were collected and stained with antibodies or isotypes for 30 min on ice. Stained cells were washed with FACS buffer (PBS with 2% FBS) followed by fixing in 4% Paraformaldehyde (PFA) for 20 min. Fixed cells were then suspended in FACS buffer and analyzed on the LSR II XW4400 workstation (BD Biosciences).

### Animal studies

4-5-week-old female BALB/c nu/nu athymic nude mice (Envigo, Madison, WI) were implanted with 3×10^6^ MDA-MB-231 cells transfected with empty vector (*n* = 17) or LSD2 expression vector (*n* = 16) into the mammary fat pad. Tumor volumes were regularly assessed every two days by measuring 0.5 × length (mm) × width (mm) × width (mm). Mice were also weighed every two days. At the end of study, tumor or lung tissues of animals were collected and fixed with 4% paraformaldehyde. Tissues were processed into paraffin sections, and then subjected to hematoxylin-eosin (H&E) staining at the histological core facility at Magee Womens Research Institute.

### Statistical analysis

Data were represented as the mean ±SD or ±SEM of three independent experiments. Two-tailed Student’s t-test was used to determine the quantitative variables. Statistical analyses were performed using GraphPad Prism 6 (GraphPad Software Inc., La Jolla, CA). *P*-values < 0.05 were considered statistically significant for all tests.

## SUPPLEMENTARY MATERIALS FIGURES AND TABLES





## Abbreviations

KMT: histone lysine methyltransferase
KDM: histone lysine demethylases
LSD1/KDM1A: histone lysine-specific demethylase 1/1A
LSD2/KDM1B: histone lysine-specific demethylase 2/1B
TCGA: The Cancer Genome Atlas
AML: acute myeloid leukemia
DNMT: DNA methyltransferase
TNBC: triple-negative breast cancer
BCSC: breast cancer stem cell
ALDH: Aldehyde Dehydrogenase
MET: mesenchymal-epithelial transition
HDAC: histone deacetylase
NuRD: nucleosome remodeling and histone deacetylase
PCNA: proliferating cell nuclear antigen
ESC: embryonic stem cell
CSC: cancer stem cell
