# Supplementary material for: Functional characterization of lysine-specific demethylase 2 (LSD2/KDM1B) in breast cancer progression
Source: Oncotarget. 2017 Jul 19;8(47):81737–53. doi: 10.18632/oncotarget.19387 (PMC5669845; doi:10.18632/oncotarget.19387)
Supplement: Supplementary file 1 [file oncotarget-08-81737-s001.pdf]

Functional characterization of lysine-specific demethylase 2 (LSD2/KDM1B) in breast cancer progression

Supplementary Materials

Aldehyde dehydrogenase (ALDH) Detection Assay Level of ALDH in MDA-MB-231 cells was detected using AldeRed ALDH Detection Assay (MilliporeSigma, Billerica MA). All procedures were performed following manufacturer's instructions. Briefly, 2×10^5 cells were suspended in 1 ml of AldeRed Assay Buffer supplemented with Verapamil. Then 5 µl of AldeRed 588-A was added to the cell suspension in "test" tube. 500 µl of the mixture was immediately transferred to a "control" tube containing 5 µl of DEAB reagent. These two tubes were incubated at 37°C for 45 min. Cells were then pelleted by centrifugation and re-suspended in 500 µl of AldeRed Assay Buffer. Analysis of PE-Texas Red signal intensity in cells was done on the LSR II XW4400 workstation (BD Biosciences).

Microarray analysis of gene expression Total RNA samples from three independent biological replicates were extracted from MDA-MB-231 EV and LSD2-OE cells using Qiagen RNeasy kit. The array study was performed at Cancer Biomarkers Shared Facility of University of Pittsburgh Cancer Institute (UPCI) using the Affymetrix GeneChip U133A 2.0 array platform, which contains 22,000+ probes representing all functionally characterized genes in the human genome to date. The data from all of the arrays were processed as RMA files (Affymetrix Robust Multi-Array Average) in which the raw intensity data were background corrected, log2 transformed and then quantile normalized according to Affymetrix recommendations.

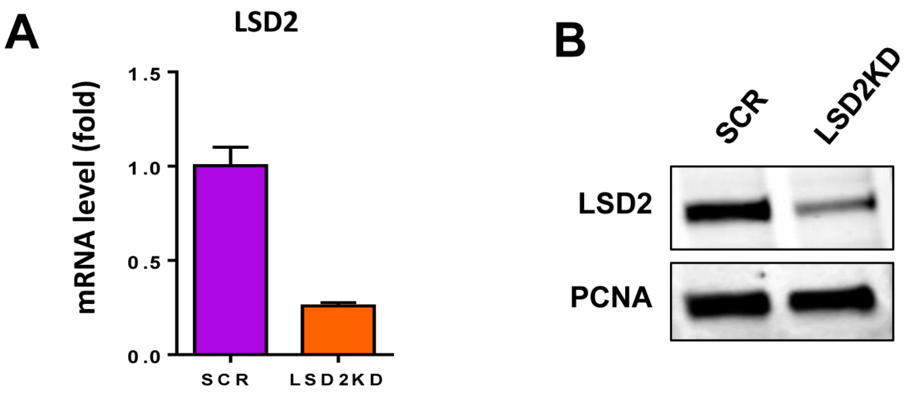

Supplementary Figure 1: Assessment of LSD2 knockdown in breast cancer cells. (A) MDA-MB-231 cells were transfected with scramble or LSD2 shRNA plasmids for 48 h followed by antibiotics selection. mRNA expression of LSD2 was measured by quantitative real-time PCR with GAPDH as an internal control. (B) MDA-MB-231 cells transfected with scramble and LSD2 shRNA plasmids were analyzed by western blots for LSD2 expression. PCNA was used as loading control.

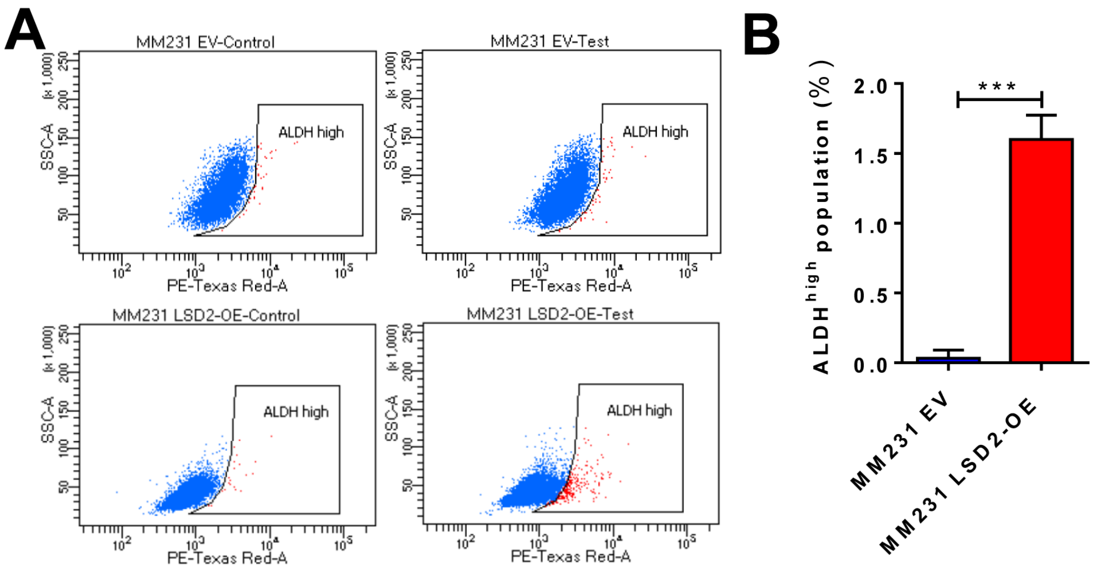

Supplementary Figure 2: LSD2 overexpression increases ALDH level in MDA-MB-231 cells. (A) ALDH levels in MDA-MB-231 EV or LSD2-OE cells were detected by flow cytometry using AldeRed ALDH Detection Assay. (B) Percentage of ALDHhigh cells was quantified. The experiment was performed three times with similar results. Columns represent means ± s.d. *** $p < 0.001$, Student's t-test.

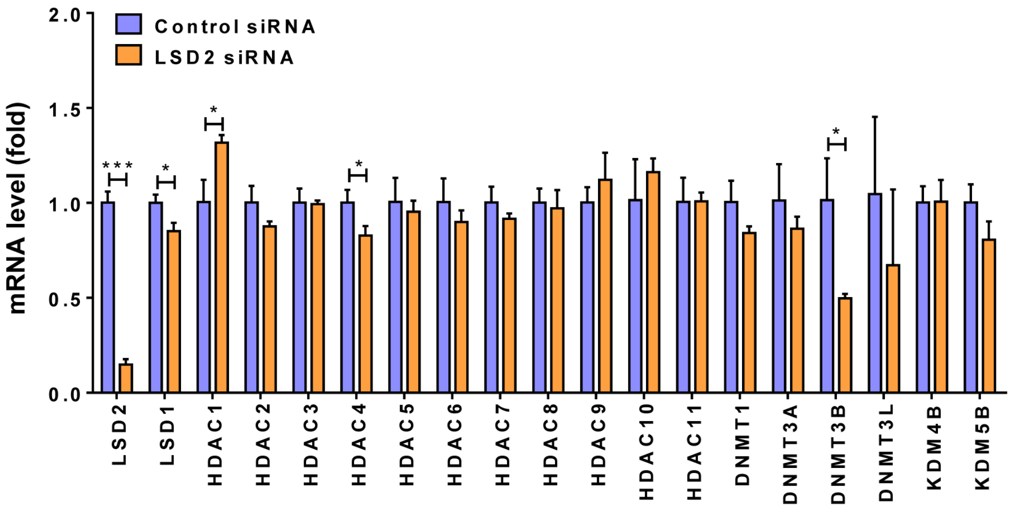

Supplementary Figure 3: Effect of LSD2 knock down by siRNA on expression of key epigenetic modifiers. mRNA expression of key epigenetic modifiers in LSD2 siRNA-treated MDA-MB-231 cells was examined by quantitative RT-PCR. GAPDH expression was used as an internal standard. Columns, means of three independent experiments mRNA expression. Bars, mean ± s.d. * $p<0.05$, *** $p<0.001$, Student's t-test.

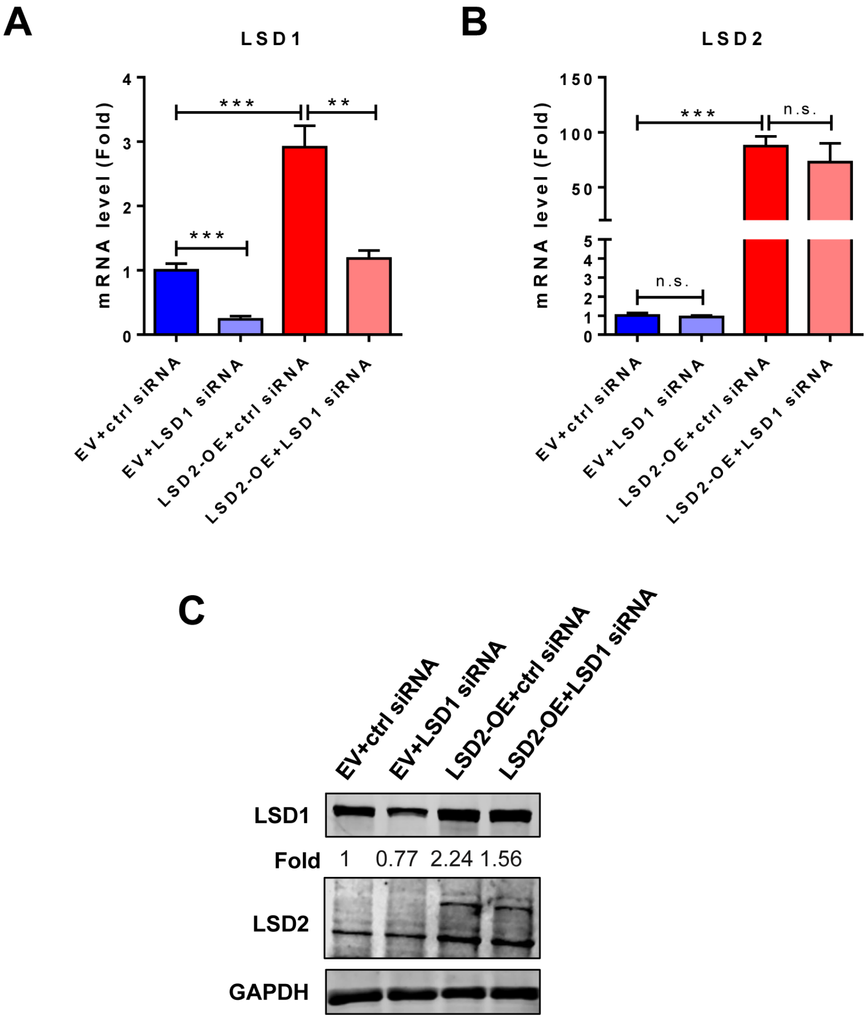

Supplementary Figure 4: Effect of LSD1 on LSD2-mediated growth in MDA-MB-231 cells. MDA-MB-231 EV or LSD2-OE cells were transfected with LSD1 siRNA for 4 days. mRNA expression of LSD1 (A) and LSD2 (B) was measured by quantitative real-time PCR with GAPDH as an internal control. (C) Protein expression of LSD1 and LSD2 was examined by western blots and GAPDH was used as loading control. Error bar represents ± s.d. from three independent experiments. n.s., not significant, ** $p<0.01$, *** $p<0.001$, Student's t-test.

For Supplementary Tables see in Supplementary Files
