# Supplementary material for: Functional characterization of lysine-specific demethylase 2 (LSD2/KDM1B) in breast cancer progression
Source: Oncotarget. 2017 Jul 19;8(47):81737–53. doi: 10.18632/oncotarget.19387 (PMC5669845; doi:10.18632/oncotarget.19387)
Supplement: Supplementary file 2 [file oncotarget-08-81737-s002.docx]

**Supplementary Table 1**: Cancer types annotation

| **Abbreviation** | **Description** | **Significant LSD2 upregulation** | ***p*-value** |
| --- | --- | --- | --- |
| BLCA | Bladder Urothelial Carcinoma | NO | 0.686104 |
| BRCA | Breast invasive carcinoma | YES | 0.001134 |
| CHOL | Cholangiocarcinoma | YES | 4.40E-05 |
| COAD | Colon adenocarcinoma | YES | 0.00467 |
| ESCA | Esophageal carcinoma | YES | 0.001134 |
| HNSC | Head and Neck squamous cell carcinoma | YES | 9.17E-07 |
| KICH | Kidney Chromophobe | NO | 7.27E-11 |
| KIRC | Kidney renal clear cell carcinoma | NO | 0.012751 |
| KIRP | Kidney renal papillary cell carcinoma | NO | 0.620305 |
| LIHC | Liver hepatocellular carcinoma | YES | 9.42E-06 |
| LUAD | Lung adenocarcinoma | NO | 0.185328 |
| LUSC | Lung squamous cell carcinoma | YES | 6.83E-05 |
| PRAD | Prostate adenocarcinoma | NO | 0.503357 |
| READ | Rectum adenocarcinoma | NO | 0.503357 |
| STAD | Stomach adenocarcinoma | YES | 2.42E-11 |
| THCA | Thyroid carcinoma | NO | 5.44E-17 |
| UCEC | Uterine Corpus Endometrial Carcinoma | NO | 0.072066 |

**Supplementary Table 2**: METABRIC dataset (Curtis Breast)

| **Dataset** | **Study name** | **Number of samples** | **Fold change** | ***p*-value** |
| --- | --- | --- | --- | --- |
| **METABRIC (Curtis Breast)** | Medullary Breast Carcinoma vs. Normal | Breast: 144  Medullary Breast Carcinoma: 32 | 1.121 | 1.56E-8 |
|  | Tubular Breast Carcinoma vs. Normal | Breast: 144  Tubular Breast Carcinoma: 67 | 1.104 | 8.96E-14 |
|  | Invasive Ductal Breast Carcinoma vs. Normal | Breast: 144  Invasive Ductal Breast Carcinoma: 1556 | 1.097 | 3.39E-26 |
|  | Invasive Ductal and Invasive Lobular Breast Carcinoma vs. Normal | Breast: 144  Invasive Ductal and Invasive Lobular Breast Carcinoma: 90 | 1.095 | 4.04E-12 |
|  | Invasive Lobular Breast Carcinoma vs. Normal | Breast: 144  Invasive Lobular Breast Carcinoma: 148 | 1.085 | 1.89E-14 |
|  | Ductal Breast Carcinoma in Situ vs. Normal | Breast: 144  Ductal Breast Carcinoma in Situ: 10 | 1.075 | 0.019 |
|  | Mucinous Breast Carcinoma vs Normal | Breast: 144  Mucinous Breast Carcinoma: 46 | 1.067 | 8.71E-7 |

**Supplementary Table 3**: Effect of LSD2 on mRNA expression of ALDH isoforms

| **ALDH isoforms** | **mRNA expression in LSD2-OE** | **Fold change LSD2-OE/EV** | ***p*-value** |
| --- | --- | --- | --- |
| ALDH1A2 | Upregulated | 106.62 | < 1e-07 |
| ALDH1B1 | Upregulated | 2.17 | 0.0000245 |
| ALDH2 | Upregulated | 1.77 | 0.0003925 |
| ALDH3B1 | Downregulated | 0.56 | 0.0000248 |
| ALDH4A1 | Upregulated | 2.51 | 0.0000064 |
| ALDH5A1 | Upregulated | 3.70 | < 1e-07 |
| ALDH6A1 | Upregulated | 2.98 | 0.0000053 |
| ALDH7A1 | Upregulated | 1.33 | 0.0320739 |
| ALDH8A1 | Upregulated | 2.65 | 0.000004 |
| ALDH18A1 | Upregulated | 1.41 | 0.0000863 |

**Supplementary Table 4**: Average of tumor volumes and *p* values

| **Tumor volume (mm^3^)** | **EV (n=17)** | | **LSD2-OE (n=16)** | | **EV vs OE** |
| --- | --- | --- | --- | --- | --- |
| **Day** | **Average** | **SEM** | **Average** | **SEM** | ***p* value** |
| 3 | 17.42 | 3.218 | 2.319 | 2.319 | 0.000699 |
| 5 | 27.64 | 2.961 | 9.424 | 3.057 | 0.000167 |
| 7 | 32.01 | 2.631 | 16.39 | 4.837 | 0.007064 |
| 9 | 37.22 | 4.398 | 19.41 | 7.892 | 0.053999 |
| 11 | 55.64 | 6.1 | 36.77 | 15.24 | 0.248803 |
| 13 | 93.83 | 11.76 | 78.13 | 29.75 | 0.619232 |
| 15 | 114.9 | 12.14 | 154.5 | 49.94 | 0.434448 |
| 17 | 175.4 | 20.28 | 267.4 | 83.4 | 0.279179 |
| 19 | 232.6 | 24.02 | 428.2 | 127.6 | 0.131042 |
| 21 | 314 | 35.47 | 634 | 174.1 | 0.073336 |
| 23 | 361 | 38.38 | 890 | 233.9 | 0.028386 |
| 25 | 480.8 | 47 | 1204 | 272.1 | 0.011232 |

**Supplementary Table 5**: Effect of LSD2-OE on expression of mesenchymal and epithelial markers

|  | **Gene** | **mRNA expression in LSD2-OE** | **Fold change LSD2-OE/EV** | **Function** |
| --- | --- | --- | --- | --- |
| **Mesenchymal Markers** | VIM | Downregulated | 0.37 | Cytoskeleton |
|  | FN1 | Downregulated | 0.11 | Cell adhesion and motility |
|  | CDH2 | Upregulated | 25.94 | Cell adhesion |
| **Epithelial Markers** | OCLN | Upregulated | 1.58 | Tight conjunction |
|  | CLDN15 | Upregulated | 1.16 |  |
|  | DSG2 | Upregulated | 1.15 |  |
|  | DSP | Upregulated | 2.31 |  |
|  | DSC2 | Upregulated | 2.40 |  |
|  | DSC3 | Upregulated | 15.63 |  |
|  | CLDN4 | Downregulated | 0.10 |  |
|  | CLDN7 | Downregulated | 0.66 |  |
|  | TJP1 | Downregulated | 0.66 |  |
|  | TJP2 | Downregulated | 0.42 |  |
|  | PARD6B | Upregulated | 1.17 | Cell polarity |
|  | LIN7A | Upregulated | 1.81 |  |
|  | SCRIB | Upregulated | 1.51 |  |
|  | DLG3 | Upregulated | 1.54 |  |
|  | LLGL1 | Upregulated | 1.22 |  |
|  | LLGL2 | Upregulated | 1.98 |  |
|  | PARD3 | Downregulated | 0.30 |  |
|  | LIN7B | Downregulated | 0.79 |  |
|  | LIN7C | Downregulated | 0.32 |  |
|  | DLG1 | Downregulated | 0.67 |  |

**Supplementary Table 6**: List of antibodies used in Western blots

| **Antibody** | **Source/Catalog Number** | **Dilution** |
| --- | --- | --- |
| LSD2 | Novus Biologicals/NBP1-90148 | 1:1000 |
| PCNA | CALBIOCHEM/NA03 | 1:1000 |
| KLF4 | Abcam/ab2543 | 1:1000 |
| Nanog | Cell signaling Technology/4903P | 1:2000 |
| OCT4 | Cell signaling Technology/2750S | 1:1000 |
| SOX2 | Cell signaling Technology/3579S | 1:1000 |
| Histone 3 H3 | Abcam/ab1792 | 1:2500 |
| LSD1 | Cell signaling Technology/2184S | 1:1000 |
| HDAC1 | Millipore/06-720 | 1:1000 |
| HDAC2 | Millipore/04-229 | 1:1000 |
| HDAC5 | Santa Cruz/sc-133225 | 1:100 |
| HDAC6 | Santa Cruz/sc-5255 | 1:100 |
| HDAC7 | Santa Cruz/sc-11489 | 1:100 |
| HDAC8 | Santa Cruz/sc-17778 | 1:100 |
| HDAC9 | Santa Cruz/sc-28732 | 1:100 |
| DNMT1 | Novus Biologicals/NB100-264 | 1:1000 |
| DNMT3A | ABGENT/AP1034a | 1:1000 |
| DNMT3B | Novus Biologicals/NB100-266A3 | 1:1000 |
| DNMT3L | Abcam/ab3493 | 1:2000 |
| GAPDH | Abcam/ab9485 | 1:2500 |
